# Growth Hormone-Secreting Pituitary Adenoma and Difficult Airway: Awake Oral Fiberoptic Intubation Approach

**DOI:** 10.7759/cureus.65889

**Published:** 2024-07-31

**Authors:** Girish Pathak, Swati Vijapurkar, Jitendra V Kalbande, Daliboina T Chandana, Gade Sandeep

**Affiliations:** 1 Anesthesiology, All India Institute of Medical Sciences, Raipur, Raipur, IND; 2 Anesthesiology and Critical Care, All India Institute of Medical Sciences, Raipur, Raipur, IND; 3 Cardiac Anesthesiology, All India Institute of Medical Sciences, Raipur, Raipur, IND

**Keywords:** regional analgesia, dexmedetomidine, acromegaly, flexible fiberoptic bronchoscopy (ffb), difficult airway management

## Abstract

Acromegaly is a rare endocrine disorder characterized by excessive growth hormone (GH) secretion, usually due to a pituitary adenoma. This condition leads to progressive somatic disfigurement, including enlarged hands, feet, and facial features, and is often associated with systemic complications such as cardiovascular disease, diabetes mellitus, and sleep apnea. Anesthesia for patients with acromegaly presents unique challenges due to the characteristic anatomical and physiological changes associated with the condition. Acromegaly, resulting from excessive GH secretion, often leads to difficult airway management, cardiovascular complications, and metabolic abnormalities. Transnasal transsphenoidal excision of pituitary adenoma is a minimally invasive surgical technique employed to remove pituitary tumors. This approach, which utilizes the nasal passages and sphenoid sinus to access the pituitary gland, offers several advantages, including reduced recovery time, minimal scarring, and lower risk of complications compared to traditional craniotomy. Awake fiberoptic intubation is one of the recommended strategies to secure an expected difficult airway such as in acromegaly. This case highlights the importance of preoperative planning and the role of an oral fiberoptic technique in managing the airway in surgeries like the transnasal approach.

## Introduction

Pituitary adenomas are tumors arising from the anterior pituitary. Pituitary adenomas are mostly benign tumors and are classified based on size. While microadenomas are less than 10 mm in size, macroadenomas are larger than 10 mm in size. A functional GH-secreting pituitary adenoma results in acromegaly, a condition due to excessive secretion of GH. This condition is characterized by excessive growth of body tissues and metabolic derangements [1]. Administration of anesthesia to a patient with acromegaly poses a challenge to the anesthetist due to the changes in the facial features and upper airway resulting in increased chances of pulmonary and cardiovascular complications. In transnasal transsphenoidal (TNTS) endoscopic excision of the tumor, the approach is via the nasal cavity to reach the pituitary gland [2].

Awake fiberoptic intubation (AFOI) has been established as the gold standard for anticipated difficult tracheal intubation [3]. AFOI is a technique that allows a flexible oral or nasal route to provide a clear visualization of the vocal cords and subsequent passage of an endotracheal tube into the trachea under direct vision in a patient who is awake and breathing spontaneously [4]. Here, we describe an oral AFOI of a patient with GH-secreting pituitary adenoma resulting in acromegaly with coarse facial features presenting as a difficult airway. 

## Case presentation

**Figure 1:**
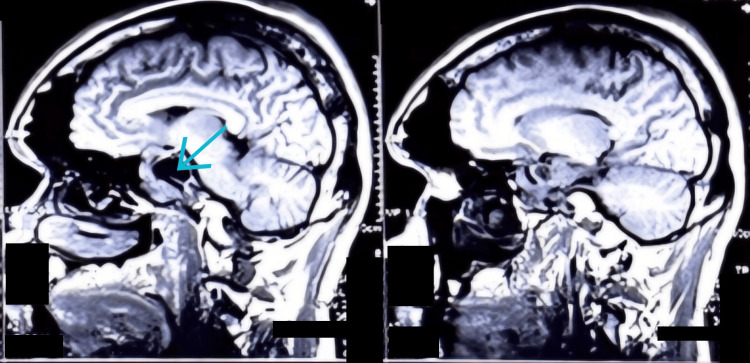
MRI showing pituitary adenoma. Arrow pointing at the necrotic/cystic center.

A 37-year-old male weighing 90 kg presented with complaints of headache for one year that was holocranial, not associated with nausea/vomiting, and coarsening of facial features since one year. The patient also had a history of hypertension for two years, which was well-controlled with medications. Magnetic resonance imaging (MRI) brain was advised by the neurosurgical department to look for any pathological condition. A heterogeneously enhancing lobulated sellar and suprasellar lesion measuring 3.5× 4.2× 3.1 cm having a large central area of necrosis/cystic area within was detected (Figure 1). Further blood investigations of the patient are shown in Table 1. A diagnosis of GH-secreting pituitary adenoma was made and the patient was planned for TNTS endoscopic excision of the tumor.

**Table 1:** Investigations specific to the condition

Parameter	Value	Reference range
Growth Hormone	73.10 ng/mL	<3 ng/mL
Insulin-like Growth Factor 1	575 ng/dL	100-284 ng/dL
Serum Cortisol	19 mcg/dL	5-23 mcg/dL
Prolactin	12 ng/mL	<20 ng/mL
Thyroid-stimulating Hormone	2.79 μIU/mL	0.5-5 μIU/mL

**Figure 2:**
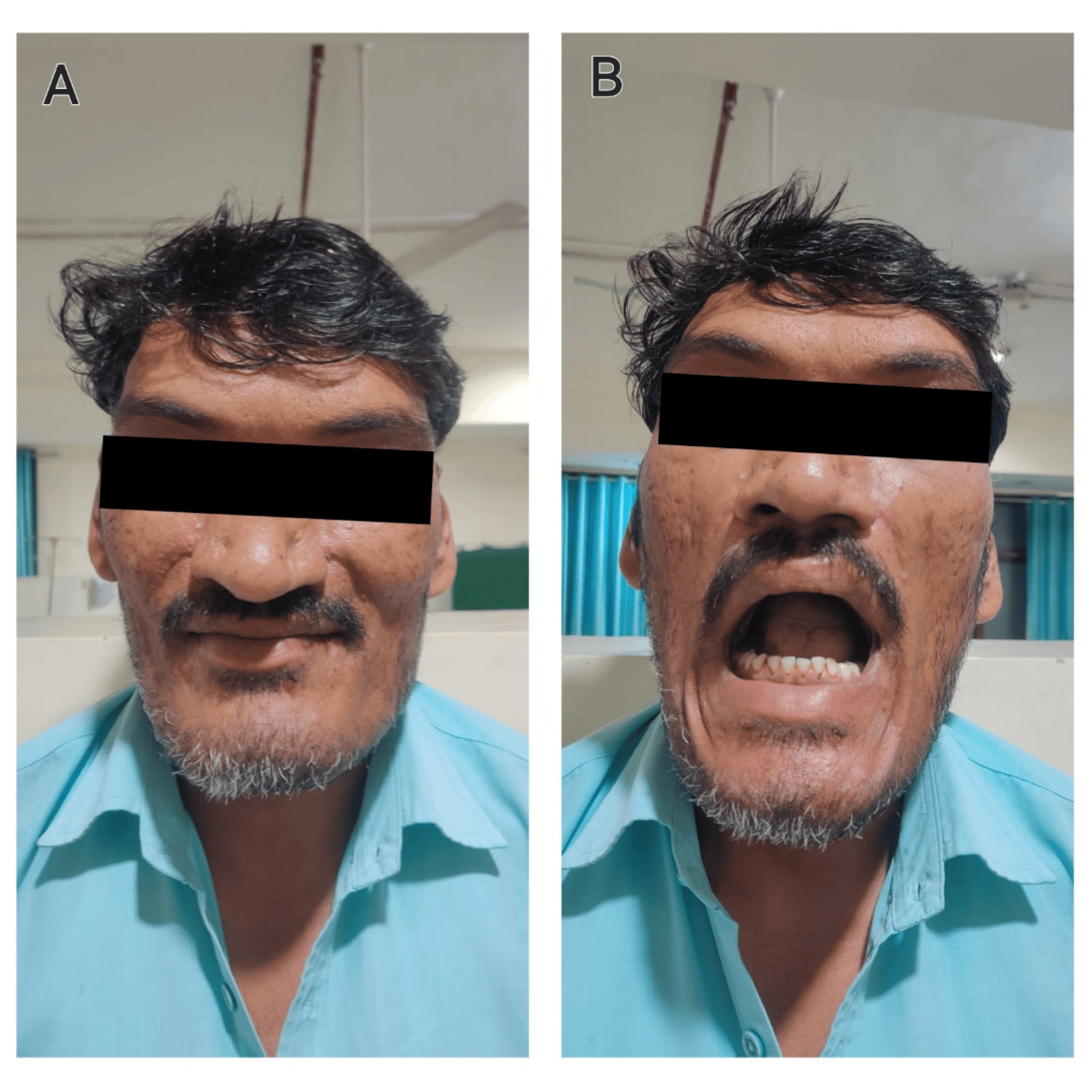
A. Image showing coarse facial features seen in the patient with enlarged nose and prognathism; B. Image showing reduced mouth opening

On pre-anesthetic checkup, he had enlarged hands and feet and a coarsening of facial features including an enlarged nose, a protruding jaw, and a restricted mouth opening (Figure 2). The cardiac evaluation showed left ventricular hypertrophy on electrocardiography and two-dimensional echocardiography had no significant finding except trivial tricuspid regurgitation with a left ventricular ejection fraction of 77%. The ophthalmic evaluation didn't reveal any visual field defects. 

Airway examination revealed Mallampati class 4, normal flexion and extension of the neck, and prognathism of the lower jaw. This was a case of an anticipated difficult airway. The plan for anesthesia was AFOI. Oral AFOI was done in this patient after adequate preparation of the oral cavity and the upper airway, as the nasal passage had to be left for surgical access. 

The airway was prepared with 4% lignocaine nebulization, 1% lignocaine viscous gargle 30 minutes before the intubation, and 10% lignocaine spray was used to anesthetize the posterior pharyngeal wall. Injection glycopyrrolate 0.2 mg was administered intravenously 15 minutes before the procedure. Xylometazoline drops were administered in the nasal cavity to reduce vascularity. Landmark-guided superior laryngeal nerve block and transtracheal injection of local anesthetic were given via a 22 gauge cannula. Before introducing the fiberoptic bronchoscope, 20 µg of injection fentanyl was given intravenously and an oropharyngeal ovassapian airway was placed. An 8.0 mm inner diameter (ID)-cuffed flexometallic endotracheal tube was loaded onto the fiberoptic bronchoscope. During the procedure, the airway was further anesthetized with 1% lignocaine via the ‘spray as you go technique’ (SAYGO) keeping in mind the total toxic dose of lignocaine. Once the fiberoptic was positioned in the trachea, the patient was induced with 100 mcg of fentanyl and 120 mg of propofol. The endotracheal tube was then slid over the bronchoscope and once the tube was inside the trachea, the fiberoptic bronchoscope was withdrawn and muscle relaxation was achieved with 9 mg of vecuronium. Anaesthesia was maintained with oxygen, air, and isoflurane at a MACage (minimum alveolar concentration for the age) of 0.9-1.0.

**Figure 3:**
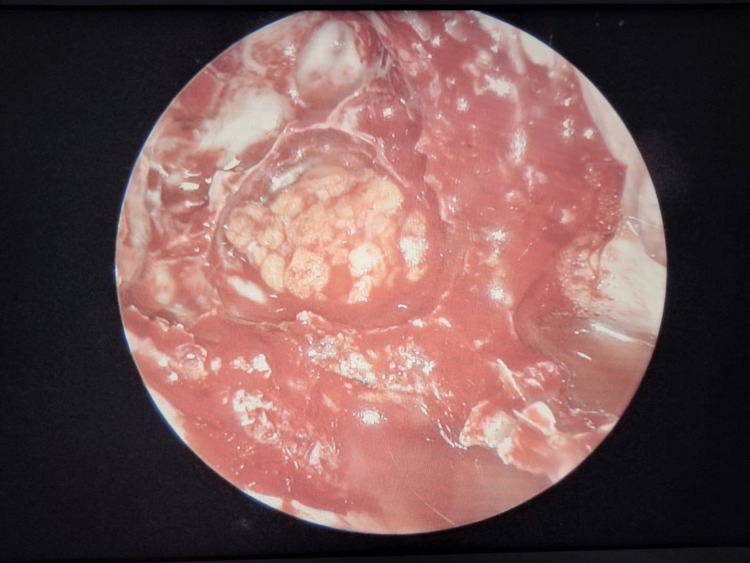
Surgical image showing the endoscopic view of the pituitary tumor.

An arterial line was secured in the left radial artery for beat-to-beat blood pressure monitoring and a central venous access was secured in the right internal jugular vein. Under ultrasound guidance, the right lateral femoral cutaneous nerve block was performed for the harvesting of fascia lata. Injection dexmedetomidine was administered at a dose of 90 mcg over 20 minutes followed by an infusion at 0.4 mcg/kg/hour titrated as per the blood pressure and surgical response. Injection tranexamic acid was administered at a dose of 10 mg/kg. Figure 3 shows the endoscopic view of the tumor. 

The surgery lasted for 160 minutes during which a total intravenous fluid of 1300 mL was administered, and a urine output of 300 mL was drained. Blood loss during the procedure was around 400 mL. Post procedure, an arterial blood gas analysis was performed to look for the electrolytes and serum osmolarity. With the arterial blood gas report being within normal limits and an uneventful intraoperative course, the patient was planned for extubation in the operating room.

On resumption of spontaneous respiratory activity, the patient was reversed with injection neostigmine 3.5 mg and injection glycopyrrolate 0.6 mg. Once the patient was awake and following commands, he was extubated and observed in the operation room for the next 10 minutes. As there was no episode of desaturation or any signs of respiratory distress, the patient was shifted to the postoperative recovery area.

In the postoperative period, the patient’s vitals, urine output, and electrolytes were constantly monitored for one hour. After attaining a modified Aldrete score of 9/10, the patient was shifted to the ward. With an uneventful course in the ward, the patient was discharged from the hospital on postoperative day 7.

## Discussion

Macroadenomas present with mass effects leading to visual disturbances due to the compression of the optic chiasma. Cranial nerve involvement of the fourth, fifth, and sixth nerves may also be present with invasive tumors [5]. Functional adenomas cause increased secretion of the hormones that are produced by the cells that they arise from [6]. 

Acromegaly is caused by excessive production of GH from the anterior pituitary. This results in the overgrowth of certain tissues that lead to coarse facial features and involvement of multiple organ systems like cardiovascular, pulmonary, rheumatic, and metabolic derangements. Coarse facial features include a prominent forehead, prognathism, macroglossia with widely spaced teeth, a large nose and lips, and thickened pharyngeal and laryngeal soft tissues. These craniofacial anatomic changes may give rise to obstructive sleep apnoea (OSA). Cardiovascular changes like acromegalic cardiomyopathy and elevated blood pressure may be seen. Dorsal kyphosis and lumbar hyperlordosis may be present. Large hands with stubby fingers, carpal tunnel syndrome, and proximal myopathy are also seen in these patients [7]. 

TNTS hypophysectomy is a minimally invasive procedure and has advanced as the standard approach in the majority of pituitary tumors for surgical resection. In TNTS excision, the tumor is approached and removed via the nasal cavity. This reduces the pressure symptoms and decreases the secretion of GH [8]. This technique produces less postoperative pain, reduces the duration of hospital stay, and avoids morbidity associated with craniotomy. The endoscope improves the visualization of the tumor and reduces the chances of residual tumor [9]. 

Anesthesia for patients with acromegaly presents several unique challenges due to the physiological changes associated with the condition. Patients with acromegaly often have enlarged facial structures, including the tongue, epiglottis, and soft tissues, leading to difficult intubation and mask ventilation. Mandibular prognathism can complicate laryngoscopy and intubation [10]. In our case, the patient had coarse facial features, prognathism, and an enlarged nose, all of them adding to a difficult airway. 

Adrenaline-soaked pledgets are inserted into the nasal cavity to reduce vascularity and blood loss. Hypertension is common in acromegaly and requires adequate management and control to avoid the hemodynamic changes and arrhythmias associated with the adrenaline-soaked pledgets and reduce blood loss [11]. 

OSA and restrictive lung disease due to skeletal abnormalities may impact intraoperative oxygenation and ventilation [12]. Monitoring of arterial blood gas lung protective strategies must be considered. Prehabilitation techniques like deep breathing exercises or incentive spirometry must be employed to prevent postoperative pulmonary complications. Metabolic derangements like insulin resistance and diabetes may occur in acromegaly with an added stress response to the surgery, which may lead to derangements in glucose values. Hence, intraoperative glucose monitoring is essential [13]. 

Preoperative assessment includes thorough airway examination, cardiac assessment with echocardiogram, blood glucose charting, and review of any medications the patient is taking and their effects on anesthesia. In the pre-anesthetic checkup, the patients must also be counseled regarding the nasal packing post surgery and that they would have to breathe via their mouth while awakening from anesthesia. Preoperatively, preparedness for a difficult airway, including the availability of advanced airway management tools (fiberoptic bronchoscope, video laryngoscope) is necessary. The technique of AFOI was similar to that in Resus Review [14].

Intraoperatively, pharmacological agents like dexmedetomidine [15] to increase the depth and attenuate stress response, and a 20-25 degree head-up position to reduce blood loss are also used. Use of regional techniques intraoperatively, like lateral femoral cutaneous nerve block, can be used during fascia lata graft harvesting for analgesia and to attenuate the stress response [16]. 

Postoperatively monitoring for respiratory complications, especially in patients with OSA, hemodynamics, urine output, and electrolytes must be done.

## Conclusions

Anesthesia management for pituitary tumor resection via the transnasal transsphenoidal approach requires meticulous preoperative planning and execution to ensure patient safety and optimal surgical outcomes. AFOI is a feasible approach for patients with a difficult airway when nasal intubation is contraindicated. Extubation must be performed after the patient is completely awake and postoperative monitoring of vitals, urine output, and electrolytes is crucial. 
